# Opportunities and challenges of computer aided diagnosis in new millennium: A bibliometric analysis from 2000 to 2023

**DOI:** 10.1097/MD.0000000000036703

**Published:** 2023-12-22

**Authors:** Di Wu, Jiachun Ni, Wenbin Fan, Qiong Jiang, Ling Wang, Li Sun, Zengjin Cai

**Affiliations:** a Department of Proctology, Yongchuan Hospital of Traditional Chinese Medicine, Chongqing Medical University, Chongqing, China; b Department of Proctology, Bishan Hospital of Traditional Chinese Medicine, Chongqing, China; c Chongqing College of Traditional Chinese Medicine, Chongqing, China; d Department of Coloproctology, Yueyang Hospital of Integrated Traditional Chinese and Western Medicine, Shanghai University of Traditional Chinese Medicine, Shanghai, China.

**Keywords:** bibliometrics, CiteSpace, computer-aided diagnosis, COVID-19, deep learning, VOSviewer

## Abstract

**Background:**

After entering the new millennium, computer-aided diagnosis (CAD) is rapidly developing as an emerging technology worldwide. Expanding the spectrum of CAD-related diseases is a possible future research trend. Nevertheless, bibliometric studies in this area have not yet been reported. This study aimed to explore the hotspots and frontiers of research on CAD from 2000 to 2023, which may provide a reference for researchers in this field.

**Methods:**

In this paper, we use bibliometrics to analyze CAD-related literature in the Web of Science database between 2000 and 2023. The scientometric softwares VOSviewer and CiteSpace were used to visually analyze the countries, institutions, authors, journals, references and keywords involved in the literature. Keywords burst analysis were utilized to further explore the current state and development trends of research on CAD.

**Results:**

A total of 13,970 publications were included in this study, with a noticeably rising annual publication trend. China and the United States are major contributors to the publication, with the United States being the dominant position in CAD research. The American research institutions, lead by the University of Chicago, are pioneers of CAD. Acharya UR, Zheng B and Chan HP are the most prolific authors. Institute of Electrical and Electronics Engineers Transactions on Medical Imaging focuses on CAD and publishes the most articles. New computer technologies related to CAD are in the forefront of attention. Currently, CAD is used extensively in breast diseases, pulmonary diseases and brain diseases.

**Conclusion:**

Expanding the spectrum of CAD-related diseases is a possible future research trend. How to overcome the lack of large sample datasets and establish a universally accepted standard for the evaluation of CAD system performance are urgent issues for CAD development and validation. In conclusion, this paper provides valuable information on the current state of CAD research and future developments.

## 1. Introduction

Computer-aided diagnosis (CAD) refers to the analysis and modeling of patient data and images with the aid of computer-related technology to assist physicians in diagnosing and selecting appropriate treatment options. The concept of CAD was first introduced in the 1960s, and before 1980, several attempts were made to correlate CAD with different diseases to improve diagnostic accuracy, but these efforts were not appreciated, probably due to the immaturity of computer technology and computer penetration.^[1–3]^ In the early 1980s, systematic research and development of CAD began at the University of Chicago,^[4]^ and in 1990, Chan et al (the University of Chicago) conducted a receiver operating characteristic curve validation of an in-house computer-aided detection system and found that computer-aided detection system could improve the accuracy of clinicians in identifying microcalcifications on mammograms, confirming the benefits of CAD for clinical diagnosis.^[5,6]^ The development and validation of CAD was then extended to different diseases and modalities. In 1998, the U.S. Food and Drug Administration approved the marketing of a commercial mammography CAD device developed by the University of Chicago and manufactured by R2 Technology Inc. which marked the beginning of the commercialization of CAD.^[7]^ As computer technology gradually matured into the new millennium, the number of CAD-related research and clinical applications skyrocketed. Statistical data show that CAD was used in approximately 92% of screening mammograms in 2016 in the U.S.^[8]^ Not only in the field of mammography, but CAD is also used in combination with computer tomography (CT) and magnetic resonance imaging (MRI) for lung nodule,^[9]^ colorectal cancer,^[10]^ and intracranial aneurysms screening.^[11]^ The rapid development of CAD makes it difficult to identify research hotspots and directions for development.

Bibliometric analysis, a form of research, in which information about publications in related field is collected and analyzed using mathematical and statistical methods, is of great significance to describe the overall development trend of the research field, the composition and interrelationship of important researchers, journals and countries, and to predict research hotspots.^[12,13]^ Currently, bibliometric analysis has been used to assess research trends in breast cancer,^[14]^ orthopedic surgery,^[15]^ stem cell,^[16]^ acupuncture,^[17]^ coronavirus^[18]^ and other related fields. Such analysis can provide a reference for clinical decision-making and guideline designation, as well as standardizing the quality of scholarship.^[19,20]^

This paper applies bibliometric analysis to assess the trend in CAD-related literature, and its distribution of author, journal, country and keyword since the new millennium, in order to provide scholars who are not yet into this field or related to the field with an insight of overall research trends, research hotspots, and a forecast of future hotspots.

## 2. Materials and methods

### 2.1. Data source and search strategy

Web of Science Core Collection database is selected as target data source. Web of Science is one of the world largest and most comprehensive scholarly information resource covering a wide range of disciplines, with over 12,000 core academic journals in the most influential fields of study, including natural sciences, engineering and technology, and biomedicine.^[21–23]^ It has been widely used in bibliometric research and data visualization.^[22]^

**Figure 1.**
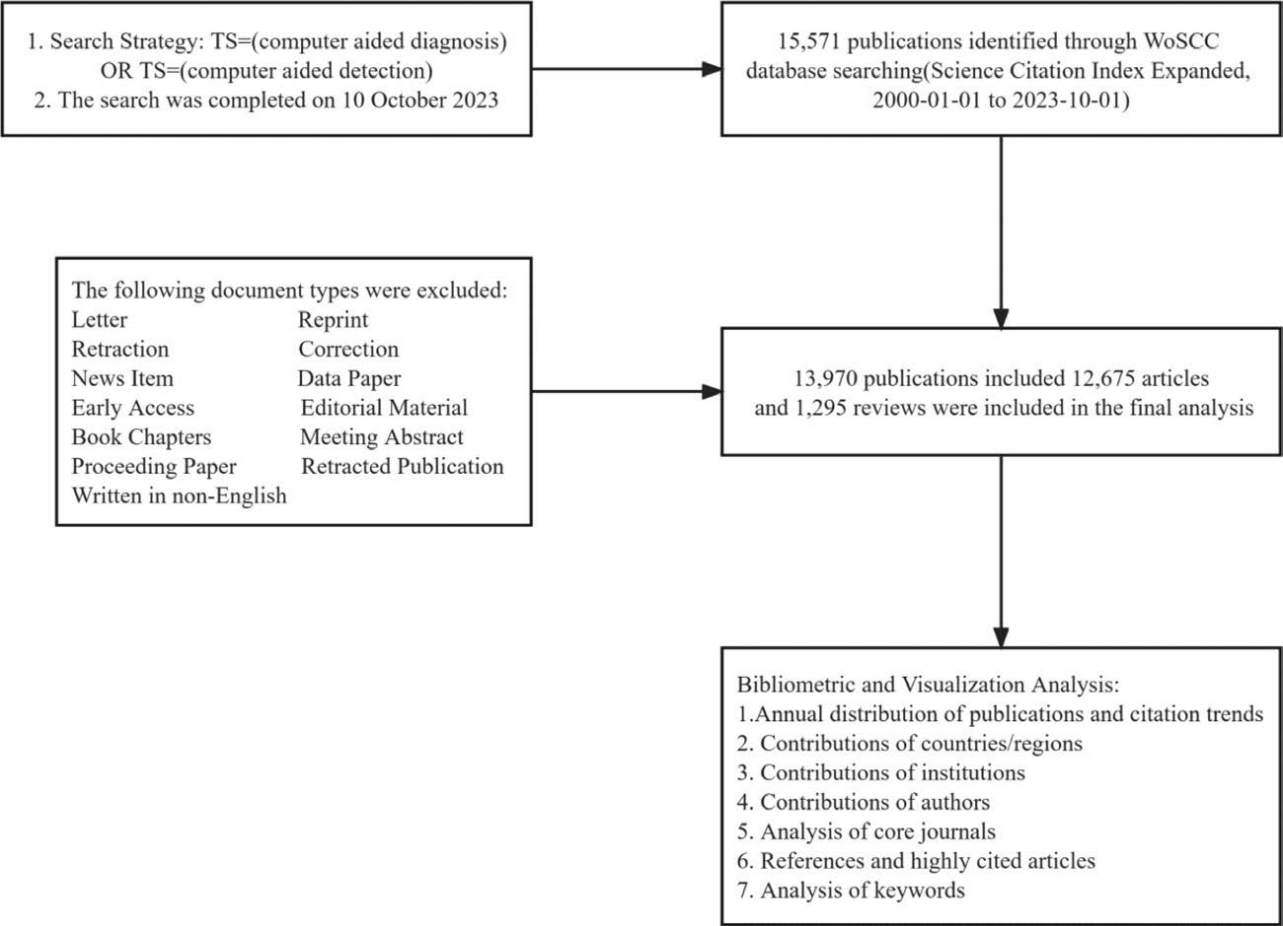
Flowchart for the selection of literature included in the study.

The relevant search information is listed as follows: Citation Index: (Science Citation Index Expanded). Search Strategy: Topic = (computer aided diagnosis) OR Topic = (computer aided detection). Document type: exclude proceeding paper, meeting abstract, early access, editorial material, letter, correction, book Chapters, data paper, news item, retracted publication, retraction, reprint. Language: English. Date range: 2000-01-01 to 2023-10-01. The retrieval was implemented and completed on October 10,2023 to avoid influence due to data update. A total of 15,571 publications were retrieved, excluding 1601 invalid records including proceeding paper, meeting abstract, early access, editorial material, letter, correction, book Chapters, data paper, news item, retracted publication, retraction, reprint, and non-English literary works. Ultimately, 13,970 publications included 12,675 articles and 1295 reviews were included as the final dataset (Fig. 1).

### 2.2. Data extraction

All data was extracted and manual proofread independently by 2 authors. In the event of a contradiction, a third author will step in and determine the final data. Extracted data include title, authors, institutions, keywords, countries/regions, year of publication, references, citation frequency. In consideration of electronic publication, the year of publication was manually calibrated to the earliest available year for the literature. When there are different names for the same country, only 1 country name is retained and relevant data will be consolidated.

### 2.3. Data analysis

Microsoft excel is used for statistics and graphing of publication amount and citation times. VOSviewer version1.6.18 is used for co-authorship, co-occurrence, citation or co-citation analysis and data visualization of countries, authors, institutions and references. One node represents 1 item and lines represent the links between different items. The thicker the line, the tighter the linkage, and the different colors represent different clusters. We use impact factor (IF) and category published by Journal Citation Reports (JCR) to evaluate journal quality.

## 3. Results

### 3.1. Annual distribution of publications and citation trends

**Figure 2.**
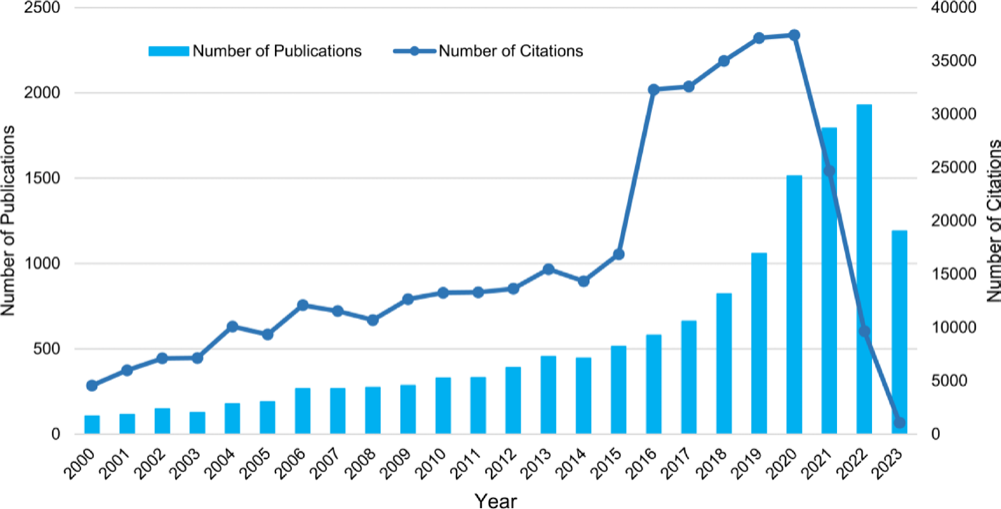
Global trend of annual publications and citations related to CAD research from 2000 to 2023. CAD = computer-aided diagnosis.

A total of 13,970 publications were included after retrieval and calibration, mainly were Articles (12,675) and Review Articles (1295). Annual distribution of publications and citation trends after manual calibration are shown in Figure 2. The overall number of publications showed an upward trend with an increase of approximately 18 times in 2022 compared to 2000. 2000 to 2014 saw a slow growth in the number of publication, fluctuating between 106 to 455. 2015 to 2022, the growth of publications and citation times entered the explosive period, with the publication amount exceeding 1500 in 2020 and citations time skyrocketing to 37,423. Up to October 10, 2023, the total citation times had reached 388,152, with an average of 27.78 citations per publication. The number of publications and citations declined in 2023, probably due to the time constraint of the search date, and it is expected that the number of publications will remain high level in 2023.

### 3.2. Contributions of countries/regions

**Figure 3.**
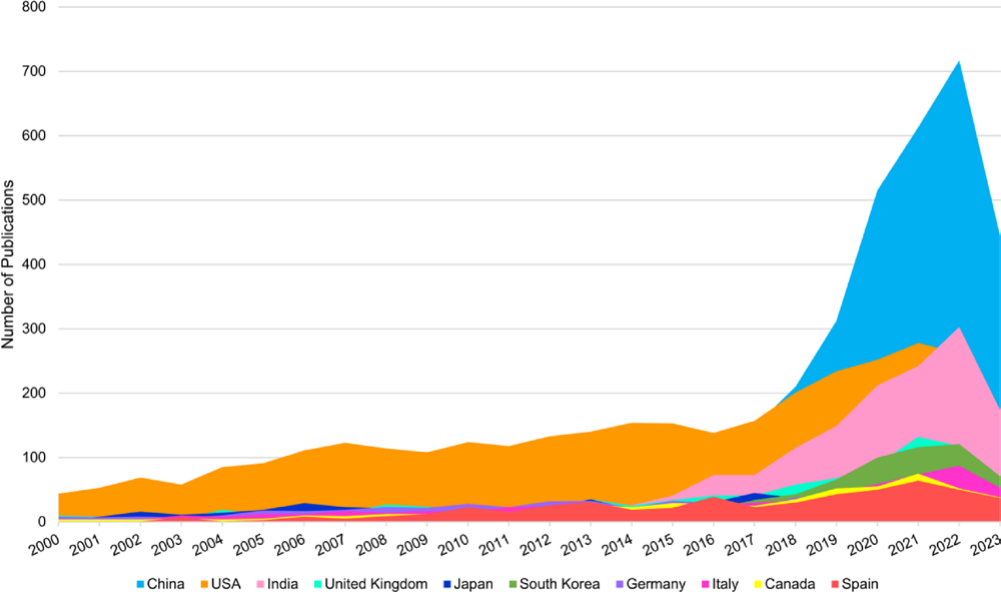
Visualization mapping of countries/regions publication. Growth trends in the publication quantity of the top 10 countries/regions CAD research from 2000 to 2023. CAD = computer-aided diagnosis.

**Figure 4.**
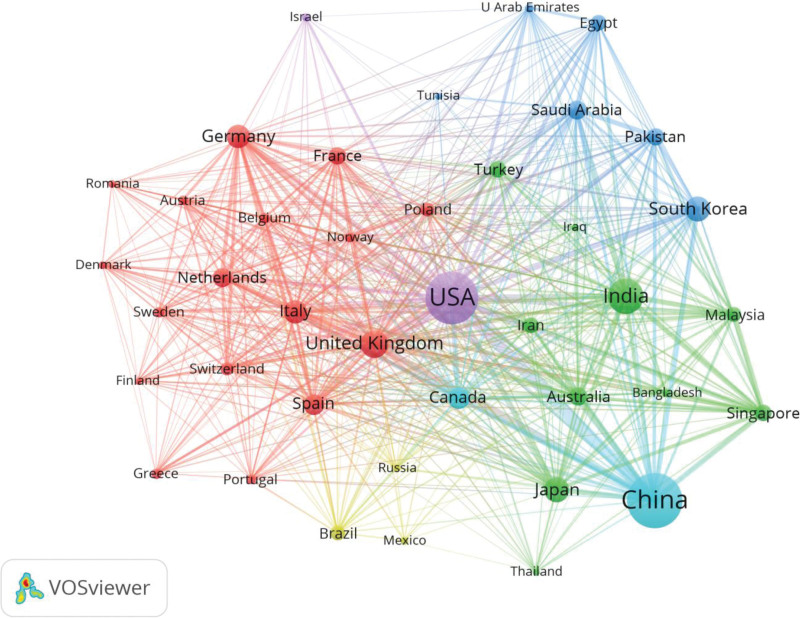
Visualization mapping of countries/regions publication. Co-authorship map of countries/regions on CAD research generated by the VOSviewer. CAD = computer-aided diagnosis.

The 13,970 publications were counted from 127 countries and regions. The top 10 countries accounted for 95.90% of the total number of publications (TP), with publications mainly from China (3684, 18.22%), the United States (3360, 16.62%), India (1493, 7.38%), England (809, 4.00%), and Japan (786, 3.89%). Among them, the United State CAD research started early, China started late but the number of publications skyrocketed after 2017, surpassing the United State as the number 1 country in terms of publication productivity per year. H-index refers to the number of authors, countries, and publications with at most h papers cited at least h times, and is a valid indicator to evaluate the productivity of publications^[24]^ (Fig. 3, Table 1). Although China has developed rapidly in CAD research in recent years, the United States still came out top of TP, average number of citations, and H-index, which highlighting the dominance of the United States in CAD research. Figure 4 is a collaboration network map of the top 40 countries by number of publications generated by VOSviewer. The line represents cooperation. The thicker the line, the stronger the cooperation. As can be seen from the figure, international cooperation is divided into 5 clusters: China (3684) and Canada (555) group; Israel and the United States (3360) group; Korea, West Asia and North Africa group: this group is mainly composed of Korea and West Asia and North Africa countries, and most of the publications are from Korea (770); Europe group: this group is mainly constituted by European countries, with the majority of publications originating from the United Kingdom (809), Germany (670), Italy (646) and Spain (520); Brazil, Mexico and Russia group.

**Table 1:** Top-10 most productive countries in CAD.

Rank	Countries	TP	Percentage	TC	CPP	H-index
1	CHINA	3684	18.22%	72,803	19.76	104
2	USA	3360	16.62%	153,146	45.58	160
3	INDIA	1493	7.38%	25,990	17.41	67
4	ENGLAND	809	4.00%	32,611	40.31	83
5	JAPAN	786	3.89%	21,702	27.61	70
6	SOUTH KOREA	770	3.81%	17,951	23.31	63
7	GERMANY	670	3.31%	21,991	32.82	67
8	ITALY	646	3.20%	18,653	28.87	67
9	CANADA	555	2.75%	22,808	41.10	68
10	SPAIN	520	2.57%	15,929	30.63	63

CAD = computer-aided diagnosis, CPP = number of citations per publication, CPP = TC/TP, TC = total number of citations of total publications, TP = total number of publications.

### 3.3. Contributions of institutions

**Figure 5.**
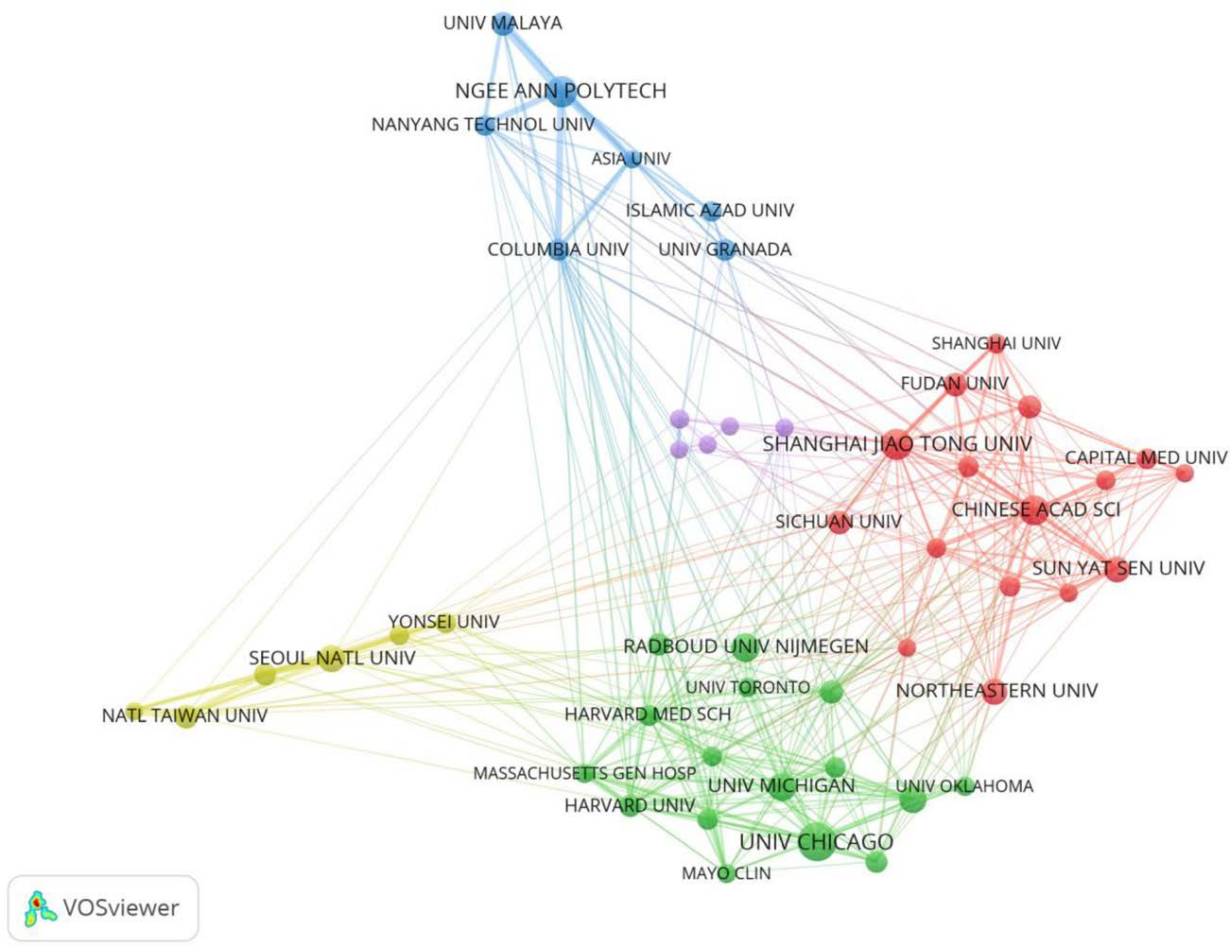
Network map of institution collaboration analysis based on VOSviewer.

**Figure 6.**
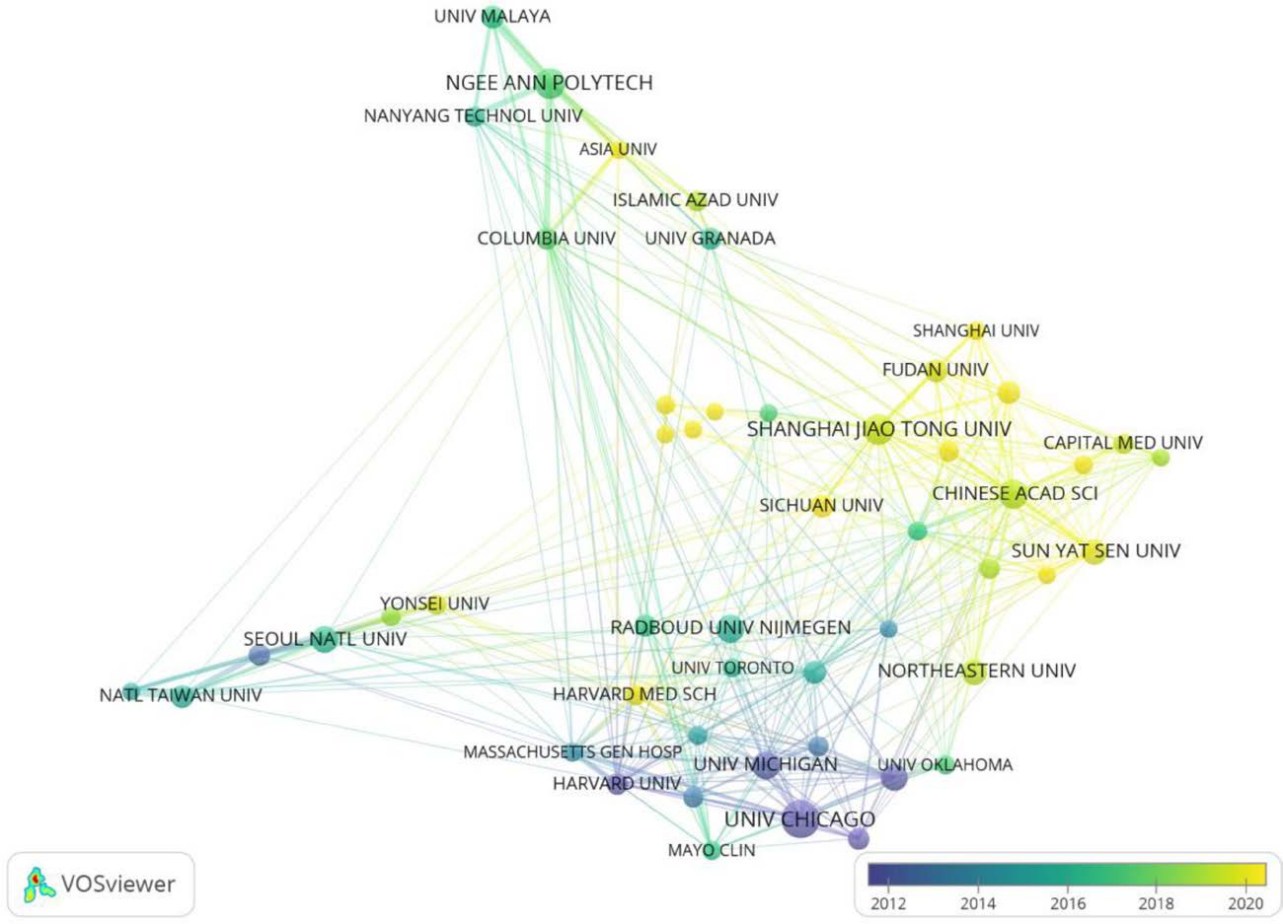
Overlay visualization of institution according to the time course based on VOSviewer.

A total of 9320 institutions contributed articles and the top 10 countries in terms of publications are listed in Table 2. The top 3 institutions are the University of Chicago (290 articles), Egyptian Knowledge Bank Ekb (273 articles) and the University of California (269 articles). The most cited were the University of Chicago (18,173) and Radboud University Nijmegen (17,906). It is worth noting that although Radboud University Nijmegen is ranked eighth, it is ranked first in terms of number of citations per publication (CPP) at 94.74. Figure 5 is a collaboration network map of the top 50 institutions according to the number of publications. As shown in the figure, the top 50 institutions are divided into 4 clusters. USA group: this group consists mainly of American research institutions led by the University of Chicago; China group: this group is mainly composed of Chinese research institutions led by Chinese Academy of Sciences and Shanghai Jiao Tong University; Korea group: this group is comprised mainly of Korean research institutions led by Seoul National University; Southeast Asia Group: This group of institutions is mainly from Southeast Asia, such as Ngee Ann Polytech, Nanyang Technological University and University of Malaya. According to Figure 6, American research institutions led by the University of Chicago are pioneers of CAD.

**Table 2:** Top-10 most productive institutions in CAD.

Rank	Institution	TP	TC	CPP	H-index
1	University of Chicago	290	18,173	62.67	70
2	Egyptian Knowledge Bank Ekb	273	5349	19.59	39
3	University of California System	269	13,384	49.75	58
4	Harvard University	246	17,719	72.03	59
5	Chinese Academy of Sciences	234	6546	27.97	41
6	University of London	210	7893	37.59	46
7	Ngee Ann Polytech	191	10,355	54.21	57
8	Radboud University Nijmegen	189	17,906	94.74	54
9	Shanghai Jiao Tong University	189	4938	26.13	34
10	Seoul National University Snu	187	4994	26.71	41

CAD = computer-aided diagnosis, CPP = number of citations per publication, CPP = TC/TP, TC = total number of citations of total publications, TP = total number of publications.

### 3.4. Contributions of authors

13,970 publications were published by 43,220 authors alone or in collaboration. The top author is Acharya UR from Ngee Ann Polytech (186), followed by Zheng B from the University of Oklahoma (previously at the University of Pittsburgh) (112), then is Chan HP from the University of Michigan (100). Most of the top 10 authors are from China. Although ranked 8th, Van Ginneken B have a CPP of 166.60 (Table 3), indicating that his research has a high impact in the field.

**Table 3:** Top-10 most prolific authors in CAD.

Rank	Author	TP	TC	CPP	H-index
1	Acharya UR	186	10,411	55.97	57
2	Zheng B	112	3498	31.23	35
3	Chan HP	100	4872	48.72	40
4	Wang Y	93	2223	23.90	25
5	Zhang Y	86	1360	15.81	21
6	Zhang J	85	2245	26.41	25
7	Doi K	79	5125	64.87	37
8	Van Ginneken B	78	12,995	166.60	43
9	Wang J	75	2200	29.33	20
10	Chang RF	74	2776	37.51	32

CAD = computer-aided diagnosis, CPP = number of citations per publication, CPP = TC/TP, TC = total number of citations of total publications, TP = total number of publications.

### 3.5. Contributions of journals

**Figure 7.**
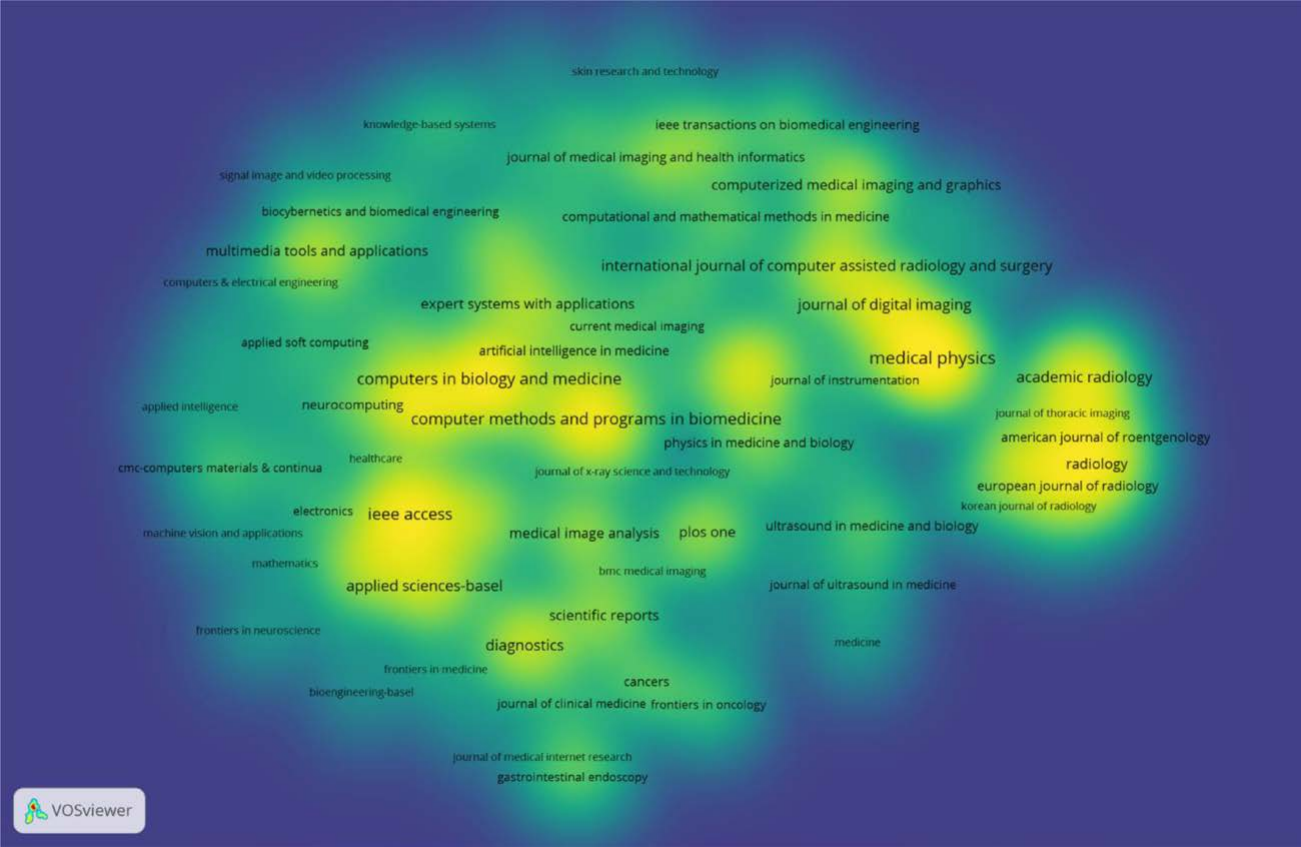
Density map of journals generated by the VOSviewer.

A total of 2046 journals published CAD-related articles. The top 10 journals published 2902 articles, accounting for approximately 20.77% of TP (Table 4). Institute of Electrical and Electronics Engineers (IEEE) Transactions on Medical Imaging is the top journal in this field with high IF and citation times. Figure 7 is a density visualization graph of the top 100 journals in terms of publication count. As can be seen, CAD-related articles are mainly published in medical or interdisciplinary journals.

**Table 4:** Top-10 leading journals in CAD.

Rank	Journal	TP	TC	H-index	JCR	IF2022
1	Medical Physics	433	17,298	70	Q2	3.8
2	Computers in Biology and Medicine	345	11,082	55	Q1	7.7
3	IEEE Access	341	5660	37	Q2	3.9
4	Computer Methods and Programs in Biomedicine	330	10,440	54	Q1	6.1
5	Biomedical Signal Processing and Control	289	4240	35	Q2	5.1
6	Academic Radiology	260	8353	48	Q1	4.8
7	IEEE Transactions on Medical Imaging	249	24,961	79	Q1	10.6
8	Journal of Digital Imaging	241	5211	38	Q1	4.4
9	Diagnostics	211	1465	20	Q2	3.6
10	Applied Sciences Basel	203	2052	22	Q3	2.7

CAD = computer-aided diagnosis, CPP = number of citations per publication, CPP = TC/TP, IEEE = Institute of Electrical and Electronics Engineers, JCR = Journal Citation Reports Category, TC = total number of citations of total publications, TP = total number of publications.

### 3.6. References and highly cited articles

The total number of citations for CAD-related articles is 45,556. The most cited article was “ImageNet classification with deep convolutional neural networks” by Krizhevsky et al in 2017, which had 810 citations. The paper proposed a large, deep convolutional neural network (CNN) to classify images,^[25]^ which has been used in CT or MRI image analysis, medical modeling and prognosis prediction for a variety of diseases^[26–28]^(Table 5). Table 6 lists the top 10 most cited articles, with 4 reviews and 6 articles.

**Table 5:** Top-10 most cited references in CAD.

Rank	Title	Yr	Resource	First author	TC
1	ImageNet classification with deep convolutional neural networks.	2017	Communications of the ACM	Krizhevsky A	810
2	A survey on deep learning in medical image analysis.	2017	Medical Image Analysis	Litjens G	489
3	Deep Residual learning for image recognition.	2016	IEEE Conference on Computer Vision and Pattern Recognition	He KM	449
4	Dermatologist-level classification of skin cancer with deep neural networks.	2017	Nature	Esteva A	374
5	Densely connected convolutional networks.	2017	IEEE Conference on Computer Vision and Pattern Recognition	Huang G	343
6	Global Cancer Statistics 2018: GLOBOCAN estimates of incidence and mortality worldwide for 36 cancers in 185 countries.	2018	CA: A Cancer Journal for Clinicians	Bray F	336
7	Very deep convolutional Networks for large-scale Image recognition	2014	Conference paper at ICLR 2015	Simonyan K	323
8	U-Net: convolutional networks for biomedical image segmentation.	2015	Medical Image Computing and Computer-Assisted Intervention	Ronneberger O	293
9	Deep convolutional neural networks for computer-aided detection: CNN architectures, dataset characteristics and transfer learning.	2016	IEEE Transactions on Medical Imaging	Shin HC	281
10	Deep residual learning for image recognition.	2016	IEEE Conference on Computer Vision and Pattern Recognition	Kaiming He	255

CAD = computer-aided diagnosis, CNN = deep convolutional neural network, ICLR = International Conference on Learning Representations, IEEE = Institute of Electrical and Electronics Engineers, TC = total number of citations of total publications.

**Table 6:** Top-10 most cited paper in CAD.

Rank	Title	Yr	Resource	First author	TC
1	A survey on deep learning in medical image analysis	2017	Medical Image Analysis	Litjens G	5918
2	Radiomics: Images Are More than pictures, they are data	2016	Radiology	Gillies RJ	4271
3	Deep convolutional neural networks for computer-aided detection: CNN architectures, dataset characteristics and transfer learning	2016	IEEE Transactions On Medical Imaging	Shin HC	3042
4	PI-RADS prostate imaging—Reporting and data system: 2015, Version 2	2016	European Urology	Weinreb JC	1999
5	Decision aids for people facing health treatment or screening decisions	2009	Cochrane Database Of Systematic Reviews	O’Connor AM	1884
6	Convolutional neural networks for medical image analysis: Full training or fine tuning?	2016	IEEE Transactions On Medical Imaging	Tajbakhsh N	1638
7	Convolutional neural networks: an overview and application in radiology	2018	Insights Into Imaging	Yamashita R	1341
8	Artificial intelligence in radiology	2018	Nature Reviews Cancer	Hosny A	1259
9	The lung image database consortium, (LIDC) and image database resource initiative (IDRI): A completed reference database of lung nodules on CT scans	2011	Medical Physics	Armato SG	1250
10	Biomarkers in cancer staging, prognosis and treatment selection	2005	Nature Reviews Cancer	Ludwig JA	1245

CAD = computer-aided diagnosis, CNN = deep convolutional neural network, CT = computer tomography, IEEE = Institute of Electrical and Electronics Engineers, TC = total number of citations of total publications.

### 3.7. Keywords analysis

**Figure 8.**
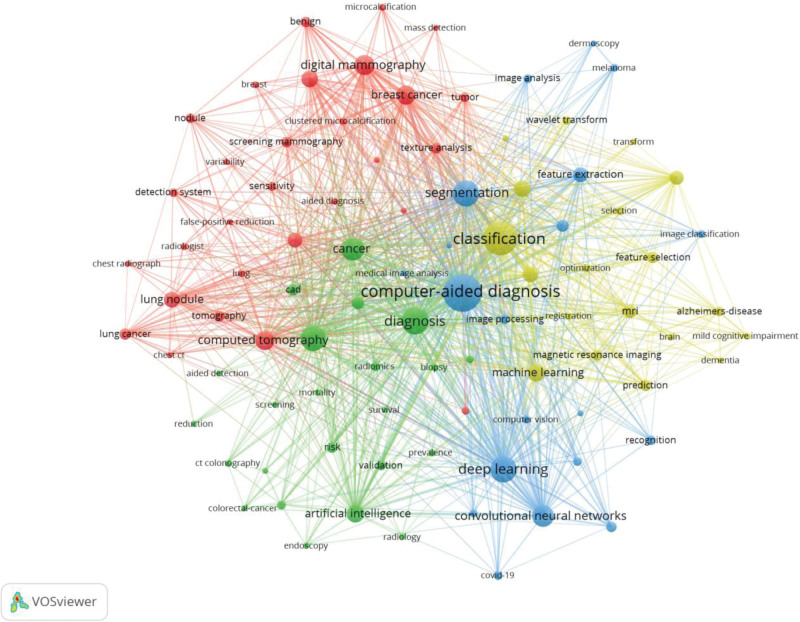
Keyword co-occurrence analysis on CAD research using the VOSviewer. CAD = computer-aided diagnosis.

**Figure 9.**
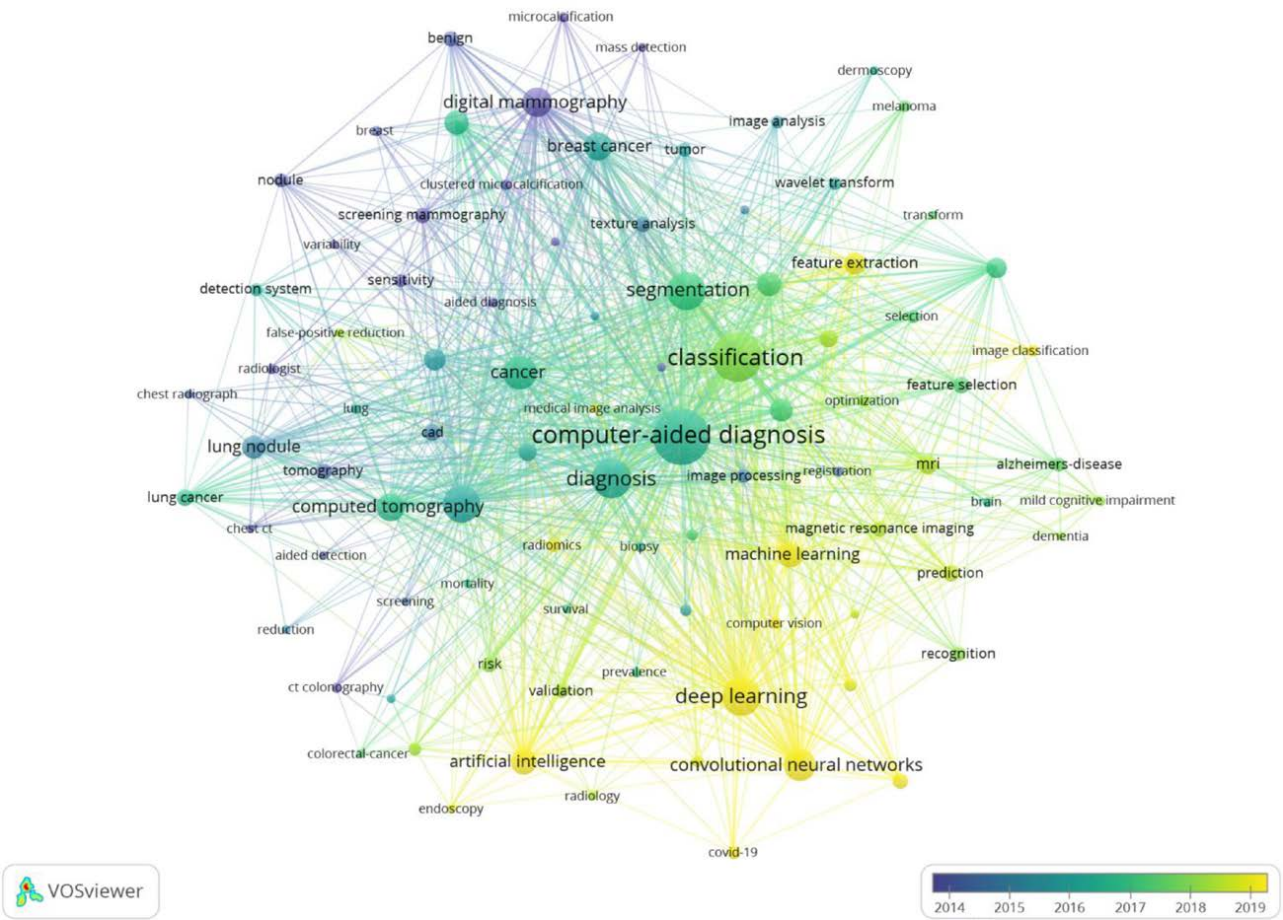
Overlay visualization of keywords according to the time course.

**Figure 10.**
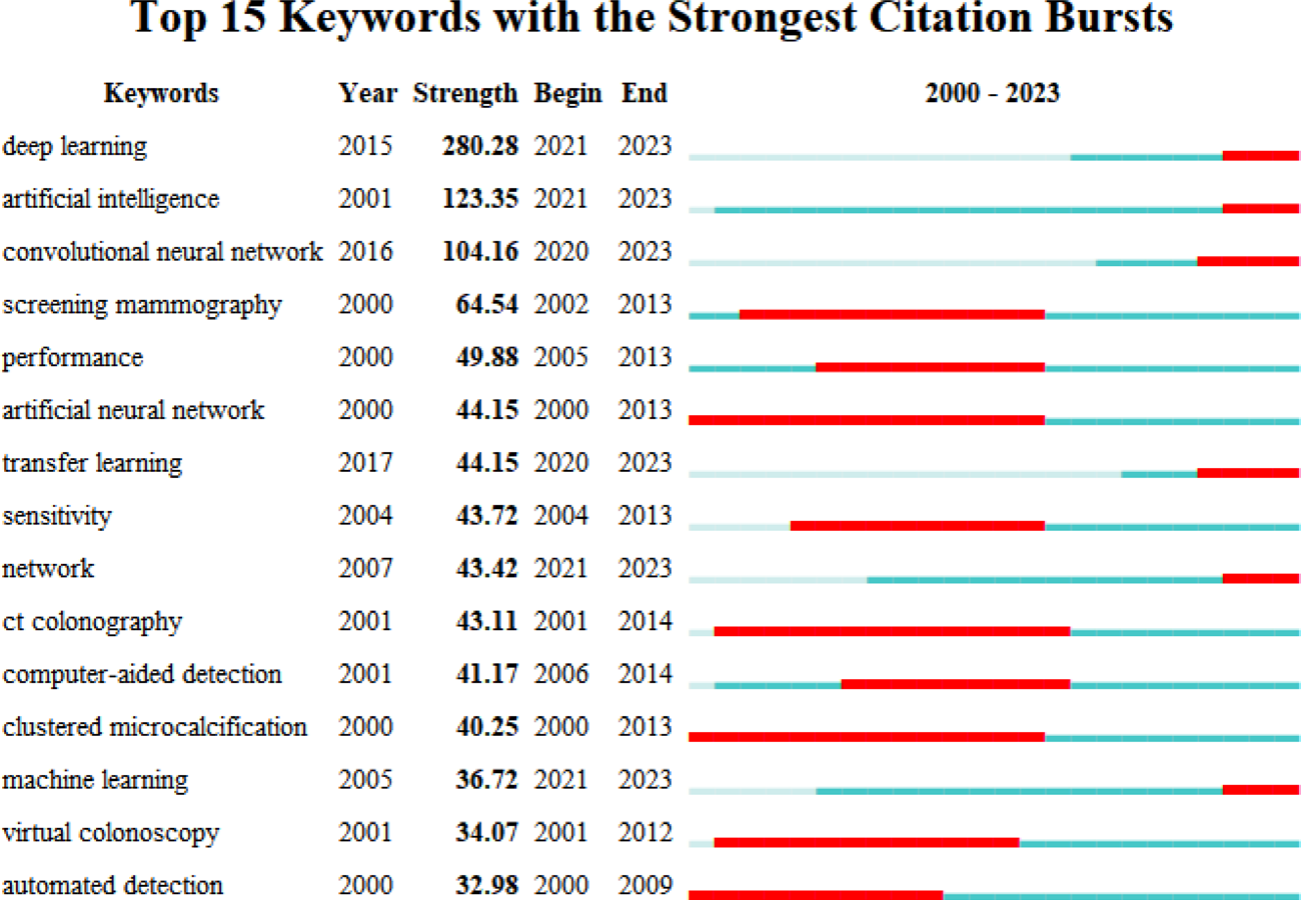
Top 15 keywords with the strongest citation bursts (sorted by brustness strength). Notes: The blue bars mean the reference had been published; the red bars mean citation burstness.

Figures 8 and 9 are co-occurrence and overlay visualized network maps of 91 keywords with more than 100 occurrences. Keyword co-occurrence analysis is one of the methods to identify prominent research hotspots in a particular field, and network map of keyword co-occurrence analysis can intuitively reflect the clustering situation and development trend of research hotspots. As is illustrated in the picture, CAD-related keywords are divided into 4 clusters: cluster I: this cluster is centered on CAD and mainly contains keywords related to the effect of CAD such as deep learning, segmentation, feature extraction, etc and new technologies; cluster II: this cluster is dominated by computer-aided detection and mainly contains keywords related to detection and prediction of diseases, such as computer-aided detection, radiomics, cancer, validation, etc; cluster III: this cluster mainly contains keywords related to new technologies in CAD and brain diseases, such as machine learning, classification, feature selection, Alzheimers-disease, dementia, etc; cluster IV: containing keywords mainly related to pulmonary diseases and breast diseases, such as computed tomography, digital mammography, breast cancer, lung nodule, etc. As can be seen from Figure 9, the yellow dots are the research hotspots of CAD in recent years, including deep learning, CNN, machine learning, artificial intelligence and feature extraction, etc. Table 7 lists the top 20 keywords in terms of frequency of occurrence. Figure 10 shows the Top 15 Keywords ranked by brustness strength generated by CiteSpace. Strength indicates how much the keyword has changed in a short period of time, with larger values representing greater variation. The top 3 keywords as ranked by brustness strength are deep learning, artificial intelligence, CNN.

**Table 7:** Top-20 most popular keywords in CAD.

Rank	Keyword	Occurrences	Rank	Keyword	Occurrences
1	Computer-aided diagnosis	3830	11	Image	985
2	Classification	2732	12	Machine learning	864
3	Deep learning	1920	13	Artificial intelligence	847
4	Diagnosis	1733	14	Feature	802
5	Computer-aided detection	1727	15	Performance	793
6	Segmentation	1470	16	Neural network	771
7	Convolutional neural network	1147	17	Algorithm	659
8	Cancer	1138	18	Feature extraction	627
9	System	1107	19	Model	616
10	Breast cancer	1014	20	Pulmonary nodule	578

CAD = computer-aided diagnosis.

## 4. Discussion

### 4.1. General information

The rapid development of computer technology into the new millennium led to the rise of CAD, and meanwhile brought it with both opportunities and challenges. Our bibliometric analysis of CAD related publication in Web of Science Core Collection database from 2000 to 2023 yielded some interesting and valuable results. Firstly, there is a general upward trend in the annual output of CAD-related articles between 2000 and 2023. After 2016, the annual number of published articles presented a sharp increase, which may be related to the highly cited articles around 2016. It is worth noting that the majority of the top 10 cited publications were published between 2016 and 2017. The most cited of these is a review by Litjens, a scholar from Radboud University Nijmegen. This review includes 308 articles on deep learning in medical imaging, summarizing the application of deep learning to medical imaging analysis, identifying current problems and suggesting possible solutions, which is very informative.^[29]^ Focusing on CNN, Shin HC and Tajbakhsh N from America show that deep CNN architectures can improve the limitations of inadequate training datasets meanwhile using pre-trained CNNs with sufficient fine-tuning perform better than or comparable to CNNs trained from scratch, but requires a smaller training set.^[30,31]^ Radiomics aims to derive quantitative, actionable insights from conventional medical imaging modalities such as CT, MRI, etc. The goal is to construct a model that evaluates clinical outcomes, encompassing diagnostic, prognostic, or predictive aspects, enabling precise identification and characterization of pathological entities.^[32]^ In 2016, an American academic Gillies published a classic review on radiomics which describes in detail the processes, challenges and future of radiomics.^[33]^ These articles all focus on new technologies for CAD and promote its rapid development.

Moreover, bibliometrics also enables visualization analysis of countries/regions, institutions, journals, authors and references, showing co-occurrence relationships and trends. The United States and China are major contributors to CAD publications and both work closely together as the main propulsions of CAD development. In line with this, the top 10 institutions in terms of publication counts are mainly from the United States and China. There is a regional concentration of collaboration between institutions, highlighting the regional superiority. In the co-authorship analysis, American scholars posted the largest number of articles, but it is of note that Van Ginneken B (Netherlands) have a higher CPP, indicating that his research has received more attention. The main research interests of the author are deep learning. Among the top 10 journals in terms of publication volume, there are 5 in JCR Q1, 4 in JCR Q2 and one in JCR Q3. Except IEEE Transactions on Medical Imaging (IF = 10.6), Computers in Biology and Medicine (IF = 7.7), Computer Methods and Programs in Biomedicine (IF = 6.1), Biomedical Signal Processing and Control (IF = 5.1), the rest are low IF journals (defined as IF < 5). Among them, IEEE Transactions on Medical Imaging is the top journal in the field of radiology and computer science and medical imaging, receiving a high level of attention and providing an important impetus to the dissemination of CAD-related articles. The references reflect the foundations of CAD research, with 2 reviews and 8 articles in the top 10 cited references, mainly focusing on the foundations of deep learning and CNN.^[25,34]^

### 4.2. Keywords and hotspots analysis

Keyword co-occurrence analysis allows the screening of high frequency keywords. Further, brustness detection can directly reflect the evolution of research hotspots and trends.^[35]^ The top 3 keywords as ranked by brustness strength are deep learning, artificial intelligence, CNN. They are research hotspots of CAD in recent years. The keywords, identified by VOSviewer and CiteSpace, can be divided into 4 clusters, which represent the different directions and frontiers of CAD development.

#### 4.2.1. Popular methods for CAD.

Cluster I is centered around CAD and contains mainly new technologies and diseases related to CAD, such as deep learning, CNN, feature extraction and COVID-19, etc. CAD is now associated extensively with medical imaging technologies such as ultrasound,^[36]^ CT^[37]^ and MRI,^[38]^ so future developments will focus more on the CAD algorithms per se. CNN is a kind of Feedforward Neural Networks with deep structure and convolution computation, which is one of the representative algorithms of deep learning.^[39]^ It is characterized by representation learning and shift-invariant classification.^[40]^ In 1988, Zhang proposed the first shift-invariant classification and applied it to medical image detection.^[40]^ Later, in 1996, Sahiner used CNN to differentiate between lumps and normal tissue in mammograms.^[41]^ At present, CNN has been used in the diagnosis of various diseases such as brain cancer,^[42]^ Alzheimer disease,^[43]^ colorectal cancer,^[44]^ urogenital cancer,^[45]^ etc. The novel coronavirus outbreak in 2019 had put enormous pressure on the public health worldwide, but it had also given CAD the opportunity to do its job. The diagnosis of COVID-19 is mainly dependent on laboratory examinations and imaging data.^[46]^ Some scholars have embarked on combining CNN with medical imaging to improve the efficiency of COVID-19 diagnosis and have achieved some progress. Ozturk et al developed a new model for automatic detection of raw chest X-ray images of COVID-19. The model was validated to have a combined detection accuracy of 92.55% for COVID-19.^[47]^ Xiao et al proposed a CNN with a parallel attention module (PAM-DenseNet) to address the discrepancy in diagnostic results caused by the different appearance, size and location of lesions in CT scans.^[48]^ It can automatically depict the infected area and reduce the workload of doctors during the outbreak. The result of the clinical trial showed that this CNN can portray the infected region with 94.29% accuracy. In the future, the algorithm can be upgraded to further improve accuracy, or the sample size of the dataset can be increased to constantly improve the reliability of the accuracy.

#### 4.2.2. Clinical applications for CAD.

The rest of the clusters are dominated by breast diseases, pulmonary diseases and brain diseases respectively, indicating that these diseases are hot spots in CAD research. In addition, CAD has been applied to the diagnosis of other diseases in recent years. Li et al combined CAD with Time-Lapsed Colposcope to classify cervical cancer and found that the accuracy of CAD classification was 78.33%, which is useful for clinical practice.^[49]^ Meng et al present a publicly available Cervical Histopathology Dataset to aid CAD research and development in cervical cancer.^[50]^ Song and his team developed a CAD-based clinically applicable system for the early diagnosis of gastric cancer, which was proven in a multicenter study to be as accurate as, or even more accurate than, the average accuracy of some pathologists and to have a stable performance.^[51]^ Hu et al published the first publicly available histopathology dataset for gastric cancer in January 2022 and validated the accuracy between different classifiers, showing the best accuracy of 96.47% with deep learning.^[52]^ The broadening of the disease spectrum in combination with CAD is the future trend. However, this faces several challenges. Firstly, most of the current CAD systems are based on CNNs, which require large sample datasets for training and validation, but the extant pool of effectively annotated samples is presently constrained, exhibiting divergence in the standards of individual sample libraries. Even if a dataset is readily available, the heavy tagging effort and the requirement for expert experience also slows down the practical availability of the dataset. Secondly, a conspicuous deficit exists in the establishment of a universally accepted standard for the evaluation of CAD system performance. Moreover, the exigencies of clinical applications mandate the identification of all signs, and in certain instances, a confluence of signs associated with multiple diseases. The discerned accuracy of CAD systems in the realm of clinical detection and diagnosis remains suboptimal, with application outcomes yet to attain the anticipated level of efficacy. Future research could focus on the creation of multinational databases, the establishment of a standardized process system for performance evaluation metrics, and artificial intelligence labeling to accelerate the development and penetration of CAD into more diseases.

## 5. Conclusion

We used bibliometric methods to analyze the characteristics of the CAD-related publications from 2000 to 2023 and have obtained some valuable information. Currently, the annual number of CAD publications and the number of citations are on the rise by the year, which indicates that CAD technology is gradually becoming mature. Meanwhile, we identified the leading countries, authors, journals and most popular articles, confirming breast diseases, pulmonary diseases and brain diseases as popular diseases of CAD. Deep learning, artificial intelligence, CNN are hot research topics in recent years. Expanding the spectrum of CAD-related diseases is a possible future research trend. How to overcome the lack of large sample datasets and establish a universally accepted standard for the evaluation of CAD system performance are urgent issues for CAD development and validation. In conclusion, this paper provides valuable information on the current state of CAD research and an outlook of future developments.

## Acknowledgments

We sincerely thank to Professor CM Chen who invented the CiteSpace and offered it for free use.

## Author contributions

**Software:** Ling Wang, Li Sun.

**Supervision:** Qiong Jiang, Zengjin Cai.

**Writing – original draft:** Di Wu, Jiachun Ni.

**Writing – review & editing:** Wenbin Fan.
